# Is Anticoagulation Discontinuation Achievable with Citrate Dialysate during HDF Sessions?

**DOI:** 10.1155/2016/9185413

**Published:** 2016-10-10

**Authors:** Thibault Dolley-Hitze, Emmanuel Oger, Didier Hamel, Marie-Laure Lombart, Isabelle Hermès

**Affiliations:** ^1^AUB Santé, Saint-Malo Dialysis Unit, 1 rue de la Marne, 35400 Saint-Malo, France; ^2^Rennes 1 University, CHU Rennes Department of Clinical Pharmacology, INSERM Pharmacoepidemiology Team CIC0203 BIOSIT, 35043 Rennes, France; ^3^Hôpital de Saint-Malo, Medical Laboratory, 35400 Saint-Malo, France

## Abstract

Citrate dialysate has been developed for few years to replace acetate and HCl concentrates. In Online Postdilution Hemodiafiltration (OL-POST-HDF), several issues are remaining concerning the possibility of stopping anticoagulation during sessions and the side effects of citrate solutions on calcium metabolism. This 1-year monocentric retrospective study included all patients exposed to citrate in OL-POST-HDF with nadroparin decrease for more than one month. Clotting events, serum calcium, PTH, hemoglobin, CRP, depuration parameters, and treatments administrated were recorded for analysis. 27 patients experienced nadroparin decrease and 5 did not receive nadroparin at the end of the study. Nadroparin decrease and withdrawal were both associated with more clotting events whereas the use of vitamin K antagonists was protective. No significant metabolic side effects were observed. Citrate dialysate does not allow anticoagulation discontinuation or decrease but has no significant side effects on mineral bone metabolism or erythropoiesis.

## 1. Introduction

Acetate is known to provide side effects such as malaise, perdialytic hypotension, cramps, or lipid and mineral bone metabolism disorders [1–3] resulting in a worse survival [4]. Consequently, citrate dialysate, known for its improved biocompatibility, has been developed for few years. Compared to acetate, citrate was described to improve depuration of small and large molecules [5–7], to induce less inflammation [5, 8–10], to be more physiological on acid-base balance [5, 11], and to improve nutritional status [12]. It results in a better clinical tolerance of the dialysis sessions [11].

The double-edged property of citrate is its ability to chelate ionized calcium generating low calcium concentration in the dialyser during dialysis session and also in the whole blood during and after dialysis time. On the one hand, citrate could be beneficial by permitting less or no heparin use to avoid circuit clotting but it could also have negative impact of mineral bone metabolism by inducing low serum calcium. Both concerns are discussed and remain uncertain.

The anticoagulation property of citrate has been used for a long time to realize regional anticoagulation of extracorporeal circuits in critical care units [13] but requires too much care to be generalized in chronic dialysis units [14]. It was recently reported that the citrate enriched dialysate could allow heparin decrease [15] and heparin withdrawal in conventional hemodialysis with low-flux membranes [16] or with heparin-grafted polyacrylonitrile membrane [17]. In OL-POST-HDF, anticoagulation withdrawal was reported in one pilot study [18] but results should be confirmed by larger studies.

Citrate dialysate can also induce hypocalcemia in chronic hemodialysed patients either in conventional low-flux hemodialysis [19] or in OL-POST-HDF [20]. In the long term, citrate use could lead to PTH upraise and consequently could weaken bone matrix.

In our center, since the 1st of May 2013, simultaneously delivering citrate dialysate, in replacement of acetate, and conducting nadroparin withdrawal for all the patients on Gambro® machines and treated with OL-POST HDF have been decided. The aim of this work is to describe this one-year experience.

## 2. Materials and Methods

### 2.1. Study Design

This is a retrospective and monocentric study conducted on chronic and stable dialyzed patients who were exposed to citrate-containing dialysate from the 1st of May 2013 to the 30th of April 2014 in our center. All patients experienced nadroparin decrease.

Circuit clotting was defined as following: partial or total loss of circuit due to the presence of clots in any part of circuit was considered as major events; presence of clots in any part of the circuit which did not reduce the dialysis duration and did not prevent a total blood restitution was considered as minor event.

A first observation period of one month with citrate exposition was applied, during which nadroparin dose did not change (same dose as with acetate-containing dialysate). Then, nadroparin was decreased by 0,1 mL per week until it could be stopped or until clotting was observed. If one major event happened, nadroparin dose increased by 0,1 mL. If a minor event happened, the dose did not change during 1 week and could be decreased again if no clots occurred.

### 2.2. Patient Selection

All patients in our center who experienced the switch from acetate dialysate to citrate-containing dialysate were included if they have been exposed for 1 month at least (until 1 year) to citrate concentrate and if they were treated with OL-POST-HDF, with at least one attempt to decrease nadroparin. Exposition to citrate concentrate was possible even if the patients were already treated by antiplatelets drugs and/or oral anticoagulants.

Exclusion criteria to citrate-containing dialysate exposition are as follows: patients with liver disease or severe respiratory insufficiency, a need for dialysate calcium concentration different from 1,50 mEq with acetate dialysate, and under the age of 18.

### 2.3. Dialysis Parameters

When patients were switched from acetate to citrate, dialysis parameters did not change. They were treated with AK200-US® or with ARTIS®. The treatment duration ranged from 210 min to 240 min. Mean blood flow rate was 350 mL/min, dialysate flow rate was 600 mL/min for all, and high-flux polysulfone membranes from 1,9 m^2^ to 2,5 m^2^ were used (TS-SL®, ELISIO®, and VITAPES®). The citrate concentrate was contained in a bag (Select Bag Citrate®, Gambro©) and the dialysate produced was composed of citric acid 1 mmol/L, no acetate, bicarbonate 35 mmol/L, calcium 1,65 mEq, magnesium 0,5 mEq, and potassium 2 mmol/L or 3 mmol/L. Sodium ranged from 135 to 140 mmol/L. Nadroparin decrease and discontinuation started between the 1st and the 3rd month after citrate use and it was not continued if one clotting event occurred during dialysis session.

### 2.4. Variables

Different variables at the beginning of the study were recorded: age, gender, time on dialysis, and cause of kidney failure.

Dialysis variables were usually collected and then extracted from Dialog7® software: effective time of session, ultrafiltration rate, convective volume, real blood volume depurated, Kt measured by ionic dialysance, clotting events and quantity of blood residues, and increased time to hemostasis.

### 2.5. Laboratory Parameters

Every month, in the mid-week dialysis, urea, hemoglobin, PTH, CRP, and serum calcium were determined.

### 2.6. Statistical Analysis

To analyze clotting events, nadroparin use was considered as a categorical variable: baseline, decrease, withdrawal, and restart. Data were modeled as a log-linear model with the Poisson variance function. The correlations between the counts were modeled as exchangeable correlations. For comparison, the correlations were also modeled as independent. The quasi-likelihood information criterion (QIC) was used for selecting regression models and working correlations (the smaller the better and a model with exchangeable correlations had smaller QIC). The difference between the log thrombotic event rates in the baseline period and the following period(s) was *β*_1_. Some further data manipulations created a log time interval variable for use as an offset.

Continuous variables were expressed as mean values ± standard deviation. The analysis of adverse events was performed with Friedman test adapted to small samples and nonparametric distribution.* p* values were considered as significant below 0.05.

## 3. Results

Twenty-eight patients were eligible for this retrospective study. The mean age was 71.3 years (±15.4 years); 20 patients were male and 8 female. All patients had native arteriovenous fistula as vascular access. The mean duration of follow-up was 6.3 months (±3.3 months) and the duration of dialysis was 3.2 years (±3.2 years). The mean dose of nadroparin before decrease was 52.4 UI/kg (±18.6 UI/kg) and the quantity of erythropoietin (EPO) injected 137 UI/kg (±144 UI/kg). Mean C-reactive protein (CRP) was 6.5 mg/L (±6.3 mg/L) and hemoglobin 11.6 g/dL (±1.0 g/dL). Initial data are summarized in Table 1. Twenty-two patients were on antiplatelet drugs and/or oral anticoagulants: 11 aspirin alone, 2 clopidogrel alone, 3 aspirin and clopidogrel, 4 vitamin K antagonists (VKA), and 2 VKA and antiplatelet. Six patients received neither antiplatelet drugs nor VKA.

A total of 2,058 dialysis sessions were analyzed. All patients experienced nadroparin decrease. Seventeen patients were nadroparin-free during at least one session, representing 639 sessions. Five patients were nadroparin-free at the end of the study period; four of them were treated with VKA; one was treated with aspirin. In one additional patient, nadroparin has been withdrawn for 7.1 months while receiving VKA but major clotting events during dialysis occurred 10 days after VKA discontinuation. During study period, 77 clotting events appeared but no hemorrhages (Table 2). By univariate analysis, both nadroparin decrease and nadroparin withdrawal increased clotting risk (Table 3). By multivariate analysis, these results were confirmed: nadroparin decrease and nadroparin withdrawal appeared as major clotting risk factors (*p* = 0.0146 and *p* = 0.0048, resp.), as well as AK200-US dialysis machine (*p* = 0.022). The use of VKA was highly protective (<0.0001) but not antiplatelet drugs. The use of ARTIS dialysis machine and high blood volume treated were also protective (*p* = 0.022 and *p* = 0.0149, resp.). The use of intravenous iron, ultrafiltration rate, hemoglobin, CRP, and type and surface of hemodiafilters did not appear as significant clotting risk factors.

**Figure 1:**
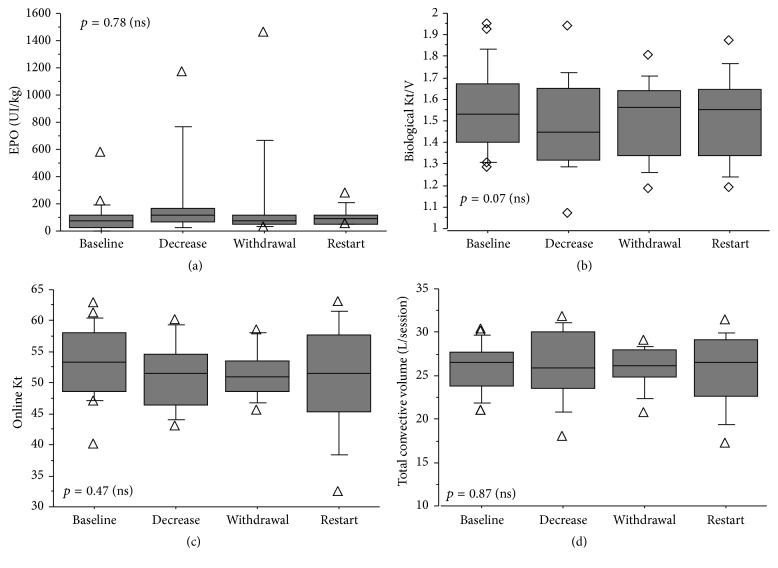
Comparison between 4 periods in nadroparin use: at baseline, during nadroparin decrease, during withdrawal, and at restart for (a) erythropoietin (EPO) use (*p* = 0.78 (ns)), (b) biological Kt/V according to Daugirdas 2 equation (*p* = 0.07 (ns)), (c) online Kt (*p* = 0.47), and (d) total convective volume (*p* = 0.87). *p* values were estimated with Friedman test.

Because clotting events happened, it was analyzed if anemia occurred or if the quantity of EPO administered increased. No significant differences were pointed out during the 4 study periods: “before nadroparin decrease,” “during nadroparin decrease,” “during nadroparin withdrawal,” and at “restart time.” Hemoglobin remained stable (*p* = 0.67 with Friedman test) as well as EPO use expressed in UI/kg (*p* = 0.78 with Friedman test). We also checked the depuration quality of small molecules and convective volumes during the 4 periods. Online Kt, biological Kt/V_sp_ according to Daugirdas 2 formula, and total convective volume did not change between the different periods (Figure 1).

**Figure 2:**
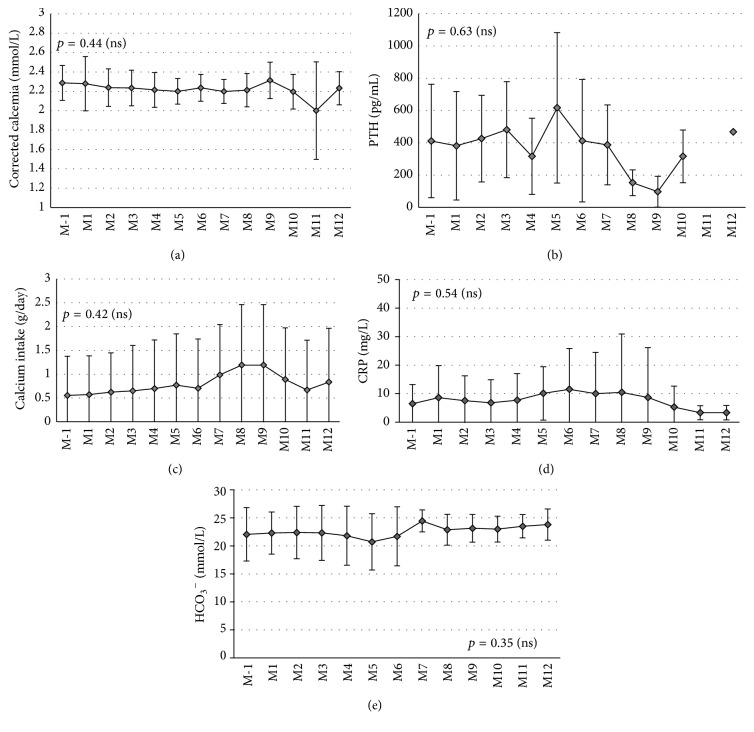
Comparison of different predialysis biological data between acetate dialysate (M-1) and citrate dialysate during the 12 following months (citrate only). No difference was observed in (a) corrected calcemia (*p* = 0.44), (b) PTH (*p* = 0.63), (c) calcium intake (*p* = 0.42), (d) CRP (*p* = 0.54), and (e) predialysis HCO_3_^−^ (*p* = 0.35). Friedman tests were applied to determine *p* value.

A 1.65 mEq calcium concentration was chosen to avoid low serum calcium and mineral bone metabolism disorders. During the first year of use, predialysis calcemia corrected with albumin remained stable. Calcemia was 2.29 mmol/L (±0.18 mmol/L) the month before citrate, 2.28 mmol/L (±0.28 mmol/L) at month 1, 2.24 mmol/L (±0.14 mmol/L) at month 6, and 2.23 mmol/L (±0.17 mmol/L) at month 12 (*p* = 0.44 with Friedman test). At the same time, quantities of oral carbonate calcium delivered did not change (0.55 g ± 0.82 the month before, 0.57 g ± 0.82 month 1, 0.70 g ± 1.03 at month 6, *p* = 0.42 with Friedman test) as well as calcium concentration in dialysate. Consequently, serum PTH concentration also did not vary during the study period: it was 411 pg/mL (±352) the month before, 381 pg/mL (±336) at month 1, and 412 pg/mL (±379) at month 6 (*p* = 0.63 with Friedman test). CRP remained stable (*p* = 0.54) as well as pre- and postdialysis serum bicarbonate (*p* = 0.35 and *p* = 0.71, resp.) (Figure 2).

## 4. Discussion

This study is the first to show clearly that both discontinuation and decrease of anticoagulation during OL-HDF-POST sessions are not achievable. If there were weak evidences to incite physicians to discontinue anticoagulation during citrate HDF sessions, heparin decrease seemed to be possible. The decrease hypothesis was supported by biological data consisting in an increase of activated partial thromboplastin time described in 2 studies [10, 21]. Some other conclusive studies conferred to the citrate dialysate the ability to reduce anticoagulation during standard hemodialysis sessions [15] and to allow anticoagulation discontinuation in OL-HDF-POST without clotting events [18].

Our results contradict these studies. The first explanation is the longer duration of our study. In previous trials, study periods were short, never exceeding few weeks, whereas we analyzed OL-HDF-POST sessions during one year. In these former works, even if clotting was not more frequent, the quantity of residual blood in dialysis circuit often increased during the period of anticoagulation decrease, suggesting a risk for clotting. A sufficient study period was required to confirm this presumption. The type of dialysis machines and the different design of circuits could also explain increased clotting risk. More frequent clotting was observed with AK200-US than with ARTIS. AK200-US represents the former generation of Gambro dialysis machine whereas ARTIS, which is the newest one, is designed with a blood circuit reducing the volume and the interface between air and blood. In Aniort's study, Fresenius 5008® machines were used with no clotting during a short period so that it can be hypothesized that circuits of 5008 machines induce very few clotting events but independently of citrate use.

We also show that VKA use is the most potent parameter to avoid anticoagulation during HDF sessions, so that physicians should try dialysis sessions without heparin by all patients receiving VKA.

Several concerns are remaining about calcium concentration in citrate dialysate and trials are contradictory. If calcium concentration in dialysate is the same as in standard concentrates, the postdialysis serum ionized calcium is always lower with citrate but not predialysis total and ionized calcium [10, 18, 21, 22]. The impact of citrate dialysate containing the same concentration of calcium on the predialysis PTH serum concentration raises questions; it remained stable in some studies [18, 21] or increased in others [20]. If calcium concentration in citrate dialysate has been increased by 0.15 mEq then postdialysis serum calcium lowers [5, 19] and if it has been increased by 0.25 mEq, serum calcium remains stable during dialysis session [8]. PTH has never been studied if calcium concentration is increased in citrate dialysate. Our long-term study should suggest the necessity for higher calcium concentration in citrate dialysate to obtain the stability of mineral bone parameters. +0.15 mEq calcium concentration in citrate dialysate could lead to stable predialysis serum calcium and serum PTH independently from oral calcium delivery. The data we present are only retrospective and should be strengthened by prospective trials.

This one-year retrospective study could suggest that citrate dialysate has no metabolic side effects, especially on acid-base balance. Nevertheless, we failed to confirm positive impact of citrate on improved depuration [20], inflammation [5, 10], and nutritional status [12].

The study has several limitations. First, it is a retrospective study that could minimize impact of citrate of mineral bone metabolism and acid-base balance. It is also to be deplored that serum citrate was not determined but this exam is not routinely performed in our center. Fortunately, former works showed that citrate does not accumulate other time [18]. We should have paid more attention to cramps and serum magnesium and it appears to be clinically relevant [20] but rarely leading to citrate discontinuation.

Finally our main result on coagulation would probably be strengthened by an awaited prospective trial of which design was recently published [23].

Long-term use of citrate dialysate in OL-POST-HDF is safe and does not seem to induce mineral bone metabolism disorders if it contains 1.65 mEq calcium more. Erythropoiesis, acid-base balance, and inflammation appear unchanged. Ultimately, citrate dialysate does not allow heparin decrease or discontinuation. VKA are still remaining to be the more potent protecting factor against clotting.

## Figures and Tables

**Table 1:** Baseline demographic and clinical characteristics.

Numbers of patients	
28	
Age (years)	
71.3 (±15.4)	
Men/women	
20 (71.4%)/8 (28.6%)	
Time on dialysis (years)	
3.2 (±3.2)	
Causal nephropathy	
12 vascular and/or diabetic nephropathy	
5 interstitial or obstructive nephropathy	
4 glomerular disease	
3 undetermined	
2 anticalcineurin nephropathy	
2 polycystic kidney disease	
Nadroparine per session (UI/kg)	
52.4 UI/kg (±18.6)	
Erythropoietin per session (UI/kg)	
137 UI/kg (±144)	
Vitamin K antagonists (VKA)/antiplatelet	
6 no treatment	
11 aspirin alone	
2 clopidogrel alone	
3 aspirin and clopidogrel	
4 VKA	
2 VKA and antiplatelet	
CRP (mg/L)	
6.9 (±6.5)	
Hemoglobin (g/dL)	
11.6 (±1.0)	

**Table 2:** Nadroparin use and clotting episodes description.

	Months on citrate (number of sessions)	Nadroparin Dose max (UI)	Nadroparin Dose min (UI)	Nadroparin Last dose (UI)	Clotting episodes	VKA	Clopidogrel	Aspirin
Patient 1	3,2 (38)	3800	950	950	1 at 950 UI	No	Yes	Yes
Patient 2	4,5 (58)	4750	0	4750	1 at 0	No	No	Yes
					8–950 to 3800 UI			
					1 at 4750 UI			
Patient 3	11,7 (139)	2850	0	1900	3 at 0 UI	No	No	Yes
Patient 4	4,1 (55)	2850	0	950	2 at 0 UI	No	No	Yes
Patient 5	2,6 (34)	2850	0	950	3 at 0 UI	No	No	Yes
Patient 6	11,6 (143)	2850	0	1900	2 at 0 UI	No	No	No
					1 at 950 UI			
Patient 7	10,2 (126)	3800	0	4750	2 at 0 UI^*∗*^	Yes^*∗*^	No	No
					4–950 to 1900 UI			
					1 at 3800			
Patient 8	6,8 (88)	1900	950	950	0	Yes	No	No
Patient 9	7 (87)	2850	0	950	1 at 0 UI	No	No	No
					1 at 950 UI			
Patient 10	3,0 (41)	3800	2750	2750	1 at 2750 UI	No	No	Yes
Patient 11	5,7 (74)	5700	0	1900	2 at 0	No	No	Yes
					3 at 1900 UI			
					1 at 5700 UI			
Patient 12	11,3 (144)	2850	0	**0**	0	Yes	No	No
Patient 13	11,6 (147)	4750	0	**0**	1 at 0 UI	No	No	Yes
Patient 14	5,2 (58)	1900	0	**0**	0	Yes	No	Yes
Patient 15	7,1 (88)	3800	0	950	1 at 0 UI	No	No	Yes
Patient 16	3,9 (45)	3800	0	950	2 at 0 UI	No	No	Yes
Patient 17	4,9 (44)	2850	0	950	2 at 0 UI	No	No	Yes
Patient 18	1,8 (23)	4750	3800	4750	9 at 3800 UI	No	No	No
Patient 19	9,5 (120)	1900	0	**0**	0	Yes	No	Yes
Patient 20	6,8 (76)	3800	950	950	1 at 1900 UI	No	No	No
					3 at 2850 UI			
Patient 21	5,2 (68)	7600	4750	5700	1 at 4750 UI	No	No	Yes
					1 at 5700 UI			
					1 at 7600 UI			
Patient 22	1,9 (17)	5700	950	1900	2 at 950 UI	No	Yes	Yes
					1 at 1900 UI			
Patient 23	5 (51)	3800	950	950	1 at 3800 UI	No	Yes	No
					3 at 2850 UI			
					1 at 950 UI			
Patient 24	11,5 (146)	3800	0	950	3 at 0 UI	No	Yes	No
					2 at 950 UI			
Patient 25	2,7 (35)	1900	0	**0**	0	Yes	No	No
Patient 26	5,8 (73)	2750	1900	2750	1 at 1900 UI	No	Yes	No
					4 at 2750 UI			
Patient 27	3,2 (40)	3800	0	1900	1 at 0 UI	No	No	Yes
Patient 28	1,7 (36)	2750	0	950	1 at 0 UI	No	No	No
					1 at 950 UI			

^*∗*^No nadroparin during 7.1 months in this patient treated with VKA. When VKA was stopped, circuits clotting occurred resulting in nadroparin restart.

**Table 3:** Association of dialysis circuit coagulation with treatment characteristics.

Parameters	Univariate analysis		Multivariate analysis		
	Estimation (±SD)	*p* value	Estimation (±SD)	*p* value	RR (95% CI)
Nadroparine quantity					
Baseline versus none	−1.06 ± 0.64	0.098	−1.63 ± 0.58	0.0048	0.20 (0.06–0.61)
Reduced versus none	−0.70 ± 0.43	0.11	−1.04 ± 0.43	0.0146	0.35 (0.15–0.81)
Hemodiafilter					
FX versus ELISIO	−0.37 ± 0.43	ns			
TS versus ELISIO	−0.38 ± 0.66	ns			
VITAPES versus ELISIO	−0.37 ± 0.52	ns			
Dialysis machine					
AK versus ARTIS	0.83 ± 0.38	0.027	1.97 ± 0.86	0.022	7.15 (1.33–38.4)
Vitamin K antagonists					
Present versus Absent	−4.01 ± 0.70	<0.0001	−3.12 ± 0.49	<0.0001	0.04 (0.02–0.11)
Antiplatelet drugs					
Aspirin versus none	0.06 ± 0.52	ns			
Clopidogrel versus None	0.73 ± 0.51	ns			
Association versus none	−0.62 ± 0.89	ns			
Iron sucrose (Venofer®)					
Present versus absent	−0.68 ± 0.29	0.02	−0.30 ± 0.31	0.33 (ns)	
Total blood treated (RR/10 liters)	−0.08 ± 0.02	0.0002	−0.09 ± 0.04	0.0149	0.40 (0.19–0.84)
Ultrafiltration rate	0.00 ± 0.00	ns			
Hemoglobin	−0.21 ± 0.17	ns			
CRP	0.002 ± 0.01	ns			

By univariate and multivariate analysis, the reduction and withdrawal of nadroparin are associated with increased risk for clots, as well as AK200 dialysis machine. VKA and high volume of blood treated during one session are protective. Antiplatelet drugs, venous iron, ultrafiltration rate, hemoglobin concentration, and CRP do not have significant impact on the risk of clots.
